# Macrophages: friend or foe in idiopathic pulmonary fibrosis?

**DOI:** 10.1186/s12931-018-0864-2

**Published:** 2018-09-06

**Authors:** Lei Zhang, Yi Wang, Guorao Wu, Weining Xiong, Weikuan Gu, Cong-Yi Wang

**Affiliations:** 1 The Center for Biomedical Research, Key Laboratory of Organ Transplantation, Ministry of Education and Ministry of Health, Tongji Hospital, Tongji Medical College, Huazhong University of Science and Technology, 1095 Jiefang Avenue, Wuhan, 430030 China; 2 Departments of Orthopaedic Surgery-Campbell Clinic, and Pathology, University of Tennessee Health Science Center (UTHSC), Memphis, TN 38163 USA

**Keywords:** IPF, Macrophage polarization, Classically activated (M1) macrophages, Alternatively activated (M2) macrophages, Therapeutic strategies

## Abstract

Idiopathic pulmonary fibrosis (IPF) is a prototype of lethal, chronic, progressive interstitial lung disease of unknown etiology. Over the past decade, macrophage has been recognized to play a significant role in IPF pathogenesis. Depending on the local microenvironments, macrophages can be polarized to either classically activated (M1) or alternatively activated (M2) phenotypes. In general, M1 macrophages are responsible for wound healing after alveolar epithelial injury, while M2 macrophages are designated to resolve wound healing processes or terminate inflammatory responses in the lung. IPF is a pathological consequence resulted from altered wound healing in response to persistent lung injury. In this review, we intend to summarize the current state of knowledge regarding the process of macrophage polarization and its mediators in the pathogenesis of pulmonary fibrosis. Our goal is to update the understanding of the mechanisms underlying the initiation and progression of IPF, and by which, we expect to provide help for developing effective therapeutic strategies in clinical settings.

## Background

Idiopathic pulmonary fibrosis (IPF) is the most common fibrosing lung disease with poor prognosis and no effective treatment. It is characterized by the development of excessive extracellular matrix deposition, leading to decreased static lung compliance, disrupted gas exchange, and ultimately, respiratory failure and death [1–4]. The median survival time for IPF patients is approximately 3 years after the initial diagnosis [5, 6]. Notably, the incidence of IPF rises dramatically with age and is estimated to be 3–9 cases per 100,000 people in Europe and North America [7, 8], which is slightly higher than that in Asia and South America.

It is believed that IPF is resulted from a complex interaction between multiple genetic and non-genetic risk factors (e.g., cigarette smoking and infection). However, the relative contribution of genetic and non-genetic risk factors is likely to vary among individuals [9]. Past genetic studies in familial and sporadic IPF patients have characterized that the surfactant-associated genes (*surfactant protein C*, *SFTPC*; *surfactant protein A2*, *SFTPA2*; and *ATP binding cassette member A3*, *ABCA3*) [10, 11] and the telomerase-related genes (telomerase reverse transcriptase, *TERT*; *telomerase RNA component*, *TERC*; and *regulator of telomere elongation helicase 1*, *RTEL1*) [12] are associated with IPF susceptibility. More recently, two large genome-wide association studies (GWASs) conducted in patients with sporadic and familiar IPF not only confirmed known associations with TERC, TERT, and mucin 5B gene (MUC5B), but also identified novel risk genes such as the *toll interacting protein* (*TOLLIP*) and the *signal peptide peptidase like 2C* (*SPPL2C*) [13, 14]. Of note, excess matrix accumulation is thought to be an important part in the pathological process of IPF, and its related proteins such as matrix metalloproteinase 1 (MMP1) and matrix metalloproteinase 19 (MMP19) that are strongly upregulated in IPF are proposed to be potential peripheral blood biomarkers [15, 16]. In support of this notion, a frameshift deletion (c.988delG, p.A330fs) in MMP1 and a nonsense mutation (c.T1155A, p.Y385X) in MMP19 were characterized in IPF patients [17]. Although these discoveries are exciting, how non-genetic factors trigger those genetic predisposed individuals to initiate IPF process is unfortunately remained poorly understood, which is not our focus in the current review and will be reviewed elsewhere.

Monocytes/macrophages, which originate from progenitors in the bone marrow, circulate in the peripheral blood or migrate into different tissues and constitute the foremost controllers of both innate and acquired immunity [18, 19]. In general, circulating monocytes leave the blood and migrate into tissues, where they differentiate into macrophages following exposure to different stimuli, including local growth factors, cytokines and microbial debris [20, 21]. Macrophages are critical to antigen removal by phagocytosis and the clearance of microbes, apoptotic cells and neoplastic cells [22, 23]. Furthermore, in activated immune responses, macrophages are an extremely heterogeneous population, exerting a combination of pro-inflammatory and anti-inflammatory functions [20, 24]. Typically, macrophages that mainly produce pro-inflammatory cytokines are called classically activated macrophages (M1), which can be activated either by IFN-γ or lipopolysaccharide (LPS). In contrast, macrophages that attenuate inflammation and encourage wound repair are referred to as alternatively activated macrophages (M2), which can be activated by IL-4 or IL-13 [25]. It is believed that enhanced macrophage M2 program is associated with fibrotic remodeling of internal organs, including the heart, kidneys, liver, gastrointestinal tract and lungs [26]. Therefore, in this review, we mainly discuss the current state of knowledge regarding the process of macrophage polarization and its mediators in the pathogenesis of pulmonary fibrosis. Our goal is to update the understanding of the mechanisms underlying the initiation and progression of IPF, and by which, we expect to provide help for developing effective therapeutic strategies in clinical settings.

## Origin of macrophages

Macrophages are small populations of leukocytes that play an important role in the cross-talk between innate and adaptive immunity [27]. As early as 1893, Metchnikoff first described the term “phagocytes” when he used a rose thorn to challenge a starfish [28]. He classified the phagocytes into macrophages (macro = big, phage = eater) and microphages (now better known as neutrophils), which promoted the development of a theory of phagocytosis [29, 30]. In the 1960s, several studies proposed that macrophages were solely derived from the terminal differentiation of circulating monocytes [31–33]. However, this theory has been refuted by the recent discoveries that most adult tissue-resident macrophages are seeded before birth, stem from the embryonal yolk sac and fetal liver, possess a self-renewing capacity and are maintained independent of circulating monocytes [27, 34]. In addition, the function of macrophages varies significantly based on their anatomical location, functional phenotype, morphology, as well as their gene expression profile, such as the alveolar macrophages, adipose tissue macrophages, Kupffer cells in the liver, and microglia cells in the central nervous system [26, 35].

## Pulmonary macrophages

Pulmonary macrophage populations are classified into two main categories: alveolar macrophages (AMs), which strategically reside in the alveoli, and interstitial macrophages (IMs), which are located within the lung parenchymal tissue [36, 37]. Given the fact that previous studies in macrophages were conducted in animals or human mononuclear phagocytes from blood and small tissue resections around tumors, Desch and colleagues recently collected pulmonary mononuclear phagocytes from fully intact non-diseased human lungs and identified five extravascular macrophage phagocytes based on phenotype, location, and gene expression, supporting the growing approval of myeloid cell diversity in tissues [38]. It is now clear that AMs colonize the lung tissues shortly after birth and display remarkable self-renewal properties independent of blood monocyte input in the steady-state [39–41]. In contrast, IMs are less well studied. Scott et al. demonstrated that the IM compartment contains cells derived from both yolk-sac macrophages and bone marrow-derived monocytes (BMDMs) [42]. However, the contribution of BMDMs to this population is still under investigation. Functionally, AMs are the chief effector cells of immune responses and have both proinflammatory and anti-inflammatory properties, whereas IMs play a major role in maintaining immune homeostasis in the respiratory tract and inducing immune tolerance to harmless antigens [43, 44]. Currently, a growing body of evidence supports a role for both AMs and IMs in the pathogenesis of pulmonary fibrosis.

## Macrophage polarization in IPF

**Fig. 1:**
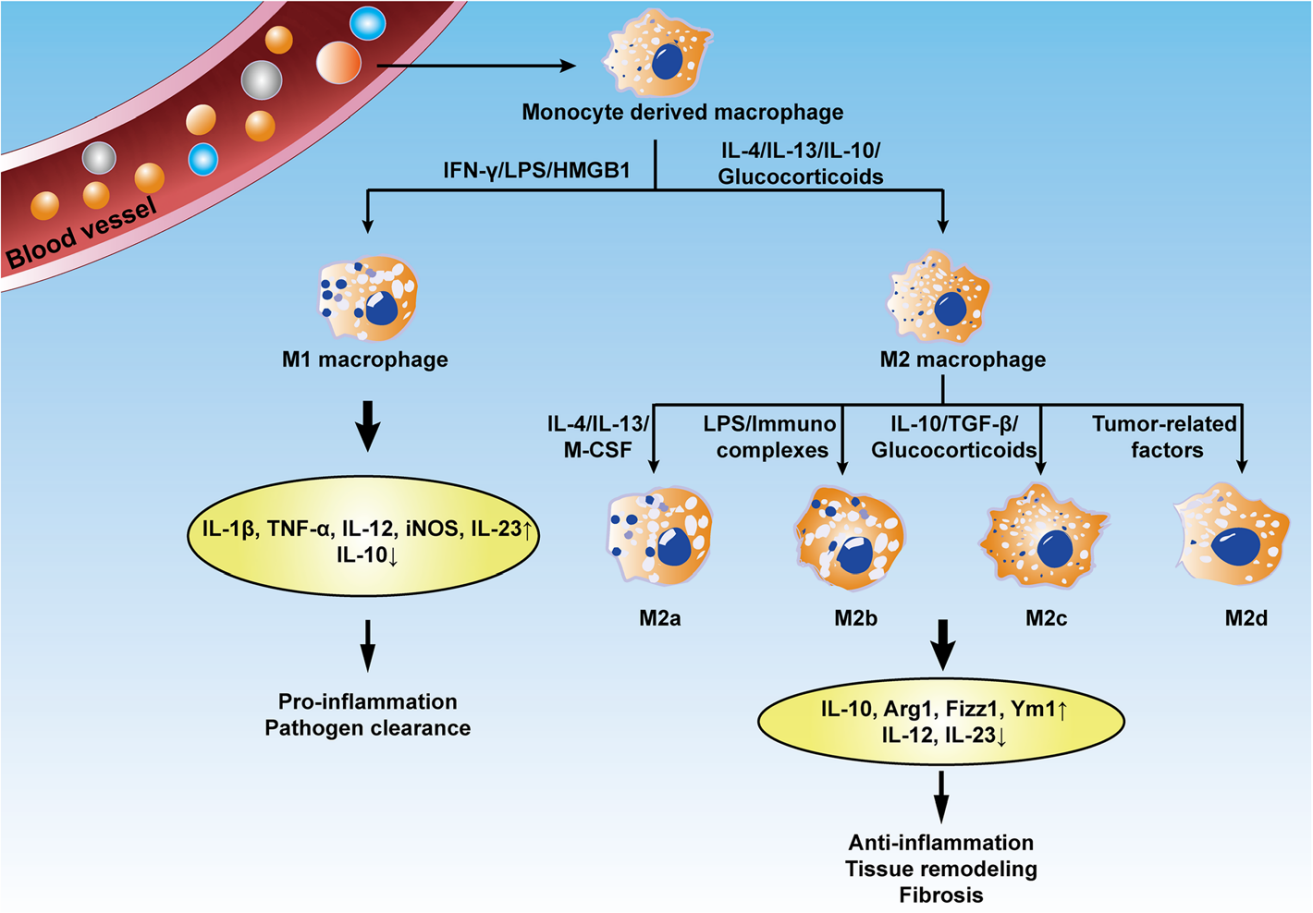
Schematic diagram of macrophage subtypes. The M1 subtype is generally considered to be proinflammatory. The M2a subtype is induced by IL-4 and IL-13, which are critical mediators of allergic inflammation. The M2b and M2c subtypes predominately participate in tissue remodeling and fibrosis. BV, blood vessel; M0, monocyte; LPS, lipopolysaccharide; IL, interleukin

Macrophages are remarkable plastic cells that can transform from one phenotype to another [42, 45]. Macrophage polarization is a dynamic process whereby macrophages manifest different functional phenotypes in response to micro-environmental stimuli and signals [25]. As the most abundant immune cells in the lungs (approximately 70% of the immune cells), macrophages play a vital role in airway remodeling in pulmonary fibrosis [43]. Beyond the AM/IM macrophage subtype delineation, pulmonary macrophages as aforementioned, can be characterized as classically activated macrophages (also termed M1) or alternatively activated macrophages (M2) [46] (Fig. 1). These macrophage subtypes differ in the expression of their cell surface markers, production of specific factors, and biological activities. Both M1 and M2 macrophages have been noted to be involved in the pathogenesis of pulmonary fibrosis, which will be discussed in detail in the following sections.

## Classically activated macrophages (M1) and IPF

M1 macrophages contribute to the host defense against pathogens by generating reactive nitric oxide (NO) via inducible nitric oxide synthase (iNOS) and releasing proinflammatory cytokines and chemokines such as IL-1β, IL-12, IL-23, CCL2 and TNF-α [47, 48]. Phenotypically, this cell type is characterized by the expression of high levels of CD80, TLR4, MHCII and CD86 [25, 49]. Therefore, M1 macrophages have robust anti-microbial and anti-tumor activities, mediate tissue damage and initiate inflammatory responses. Potent inducers such as LPS, IFN-γ, and granulocyte-macrophage colony-stimulating factor (GM-CSF), stimulate the generation of M1 macrophages, from either naïve M0 or polarized M2 macrophages [50]. Other endogenous alarmins such as oxidized low-density lipoprotein, fatty acids, caveolin-1 (Cav-1), and high-mobility group box 1 (HMGB1), are also found to induce M1 biased macrophage polarization [51–54]. Nevertheless, it still remains unknown whether they exert similar effects on pulmonary fibrosis. Of importantly note, there is evidence that the polarized M1 macrophages can be switched back to the M0 state in a cytokine-deficient medium for 12 days or re-differentiated into another cell phenotype after culture in an alternative polarizing medium [55].

In the current paradigm of IPF pathogenesis, pulmonary fibrosis progresses as a final pathological outcome of aberrant wound healing responses to persistent lung injury. Pulmonary cellular damage caused by environmental particulates, infection or mechanical damage, often result in the disruption of normal lung architecture, initiating a wound healing response [56]. At the early inflammatory stages, acute lung injury promotes an M1 phenotype under the control of transcription factor interferon regulatory factor 5 (IRF-5) with expression of high levels of iNOS and proinflammatory cytokines,which is associated with Th1 immune responses and responds to IFN-γ and toll-like receptor (TLR) ligands to maximize cytotoxic activity [57–59]. However, sustained inflammatory responses would serve as a trigger to initiate fibrotic responses in the lung. Indeed, during the course of aberrant wound healing processes, there is an early and sustained enrichment in the numbers of exudate macrophages (ExM) and their precursors, the Ly-6C^high^ monocytes [60, 61]. For example, targeted type II alveolar epithelial cell (AEC II) injury induces a modest inflammatory response that is nonetheless significantly enriched for alternatively activated and profibrotic ExM and Ly-6C^high^ monocytes. The accumulation of these nonresident cells and the development of pulmonary fibrosis in response to targeted alveolar injury is CCR2 dependent, thereby implicating ExM and Ly-6C^high^ monocytes and the CCR2/CCR2-ligand axis as potential therapeutic targets to treat or prevent fibrotic lung disease [62].

Generally, the LPS/TLR4 pathway has been considered one of the major pathways in M1 macrophage polarization [63]. For example, the LPS/TLR4 pathway activates NF-kB and IRF3 and promotes the secretion of proinflammatory cytokines (e.g., IL-6 and TNF-α) to exacerbate macrophage M1 polarization [64]. This was further supported by studies showing that the LPS/TLR4 activates STAT1 to induce M1 polarization in an MyD88-independent fashion [65]. Interestingly, deletion of both TLR2 and TLR4 increased the susceptibility to bleomycin- and radiation-induced pulmonary fibrosis [66, 67]. Similarly, IL-1R–associated kinase-M (IRAK-M), an MyD88-dependent inhibitor of TLR signaling, suppresses deleterious inflammation but may paradoxically promote fibrogenesis, where IRAK-M was noted to promote macrophage M2 program in the setting of bleomycin-induced lung injury, which drives lung fibrogenesis in an IL-13–dependent fashion [68]. Furthermore, during the evolution of radiation-induced pulmonary fibrosis, patients manifested enhanced mRNA and protein expression of iNOS within the radiation pneumonic stage, whereas the high levels of arginase 1 (Arg 1) expression occurred within the radiation-induced fibrotic phase [69]. Intriguingly, macrophage inflammatory protein-1α (MIP-1α, also known as CCL3) arises mainly from M1, while its chemotactic affinity toward M2 is stronger than it is for M1. The interaction between MIP-1α and macrophages in different activated states may play a crucial role in regulating the transition from radiation pneumonitis to radiation pulmonary fibrosis [70]. It was also noted that TLR2 was elevated in IPF patients [71], and pharmacologic inhibition of TLR2 protected mice from bleomycin induced lung injury and fibrosis [72]. Similarly, mice with macrophage specific deletion of *IRF-5* manifested fibrotic responses in adipose tissue following high-fat diet induction [73]. Collectively, those data indicate that M1 macrophage plays an essential role for wound repair, but it may also serve as a double edged sword to either trigger or prevent fibrotic responses. Nevertheless, the exact extracellular microenvironments relevant to its functional direction (e.g., in favor or against fibrotic responses) are yet to be elucidated.

## Alternatively activated macrophages (M2) and IPF

M2 macrophages could be induced by a broad array of mediators such as IL-4, IL-13, TGF-β, and IL-10, and are implicated in the aberrant wound-healing cascade during fibrosis [74]. Depending upon the specific stimulators, M2 macrophages are further subdivided into 3 subgroups, namely, M2a, M2b, and M2c [23, 45]. The M2a subtype is induced by IL-4, IL-13, fungal and helminthic infections. M2b is elicited by IL-1 receptor ligands, immune complexes, and LPS, whereas M2c is activated by IL-10, TGF-β1, and glucocorticoids. More recently, an additional M2 subset, the M2d macrophage, was identified designated by the reduced secretion of IL-12 and enhanced release of IL-10 [75] (Fig. 1). However, there is no unique surface marker to distinguish each subset of M2 macrophages, and they are characterized by low levels of MHCII, CD86, and iNOS2 but high levels of arginase-1, the family proteins chitinase-like Ym1/2 and Fizz1/RELM-α (found in inflammatory zone 1) [76, 77], and cell surface receptors such as macrophage mannose receptor, also called CD206. Analysis of over 72 non-diseased human lungs consistently indicated that CD206 is exclusively expressed on alveolar macrophages rather than on cells found in blood or in the intravascular compartment of the lung [38], which exerts an important function in the phagocytosis of M2 cells via increasing efferocytosis of invading pathogens and apoptotic cells [78]. In addition, recent studies have indicated that transcription factors and other intracellular proteins, such as tuberous sclerosis complex 1 (TSC1) [79], stress-responsive activating transcription factor 7 (ATF 7) [80], STIP1 homology and U-Box containing protein 1 (STUB1) [81], ten eleven translocation (Tet) methylcytosine dioxygenase (Tet2) [82], microRNA (MiR-511) [83], interferon regulatory factor 4 (IRF4) [84], peroxisome proliferator-activated receptor gamma (PPARγ) [85], and Krueppel-like factor 4 (KLF-4) [86] are also involved in the polarization of M2 macrophages.

During the course of IPF development and progression, the predominant infiltration of M2 macrophages in the areas of lung fibrosis acts as a vital regulator of fibrogenesis [58, 87, 88]. Generally, excessive M2-associated and Th2-driven responses are an important part of many fibrotic diseases [58, 89]. For example, alveolar M2 macrophages, which are located close in proximity to sites of pulmonary injury undergoing repair, release CCL18 to stimulate fibroblast producing collagen. On the other hand, the proximate M2 macrophages bind to collagen type I via β2-integrins and scavenger receptors, thereby further increasing their CCL18 production, which creates a self-perpetuating vicious cycle of augmented, continuous M2 macrophage activation and excessive collagen production by lung fibroblasts [90]. Indeed, upon activation, M2 macrophages can produce profibrotic mediators such as TGF-β and PDGF to induce continuous fibroblast activation and to promote myofibroblast proliferation. Similarly, IL-10 generates a Th2 microenvironment in favor of bleomycin- or helminth-induced lung fibrosis [91–93], which involves fibrocyte recruitment and M2 macrophage activation likely through the CCL2/CCR2 axis [94], leading to excessive extracellular matrix (ECM) deposition along with distorted lung tissue architecture, and ultimately resulting in pulmonary fibrosis and respiratory failure [95], although the underlying mechanisms are yet to be fully established.

In addition to Th2 cells, eosinophils, innate lymphoid type 2 cells (ILC2s), CD4^+^CD25^+^ regulatory T cells (Tregs), and mesenchymal stromal cells (MSCs) were recently reported to drive the polarization of M2 macrophages [96–99]. For example, mice infused with syngeneic CD4^+^CD25^+^ Treg cells have an increased population of CD206^+^ peritoneal macrophages, with low levels of CD80 and MHCII [100]. Interestingly, Tregs lack of *Tim-3* are noted to repress the expression of p-STAT3, thereby attenuating the induction of M2 macrophages in acute respiratory distress syndrome (ARDS)-associated pulmonary fibroproliferation [101]. Similarly, natural killer T cells (NKTs), a subset of T lymphocytes expressing membrane receptors from both T and NK lineages [102], can mediate a protective effect against fibrosis by inhibiting a Th2 response and preventing M2 macrophage polarization [103] in the bleomycin animal model.

It has been well known that IL-4 is the major inducer for macrophage M2 program by activating its two major downstream signals, JAK1/STAT6 and PI3K/AKT [88, 104]. Indeed, Guo et al. reported that elevated Gab1 and Gab2 regulate IL-4-induced macrophage polarization in bleomycin-induced fibrotic lungs, with Gab1 positively regulating AKT signaling and Gab2 positively regulating STAT6 signaling [105]. Similarly, Kral et al. noted that myeloid *PTEN* deficiency mice following bleomycin induction manifest sustained PI3K activation to enhance macrophage M2 program, leading to increased morbidity and decreased survival, although these mice exhibit impaired recruitment function [106]. Furthermore, deficiency of *shp2*, a ubiquitously expressed cytoplasmic tyrosine phosphatase, increases the sensitivity to chitin-induced M2 macrophage polarization and bleomycin-induced pulmonary fibrosis by enhancing IL-4–induced JAK1/STAT6 signaling [107]. Other than cytokines, altered adenosine metabolism was also noted to regulate macrophage M2 program. Adenosine is generated in response to cellular stress and damage, and extracellular accumulation of adenosine and subsequent activation of its receptor, ADORA2B, polarizes macrophages to a fibrotic phenotype. Therefore, mice with macrophage specific deletion of *ADORA2B* are protected from bleomycin-induced lung injury and fibrosis [108]. It was further noted that hypoxia-inducible factor 1-α (HIF-1α) enhances ADORA2B expression to regulates M2 macrophage differentiation and production of profibrotic mediators [109].

It is noteworthy that mitochondrial oxidative stress and mitochondrial turnover in alveolar macrophages are directly linked to pulmonary fibrosis [110–112], although the exact molecular mechanisms that modulate mitochondrial dynamics are currently not known. The mitochondrial calcium uniporter (MCU) is a highly selective ion channel to transport Ca^2+^ into the mitochondrial matrix for modulation of cellular metabolism [113, 114]. MCU was recently found to polarize macrophages to a profibrotic phenotype after exposure to asbestos by regulating ATP production [115]. Similarly, mitochondrial Cu,Zn-SOD accelerates the development of pulmonary fibrosis by inducing early and sustained alternative activation of macrophages through redox regulation of the Jumonji domain-containing protein 3 (Jmjd3) [116, 117]. Collectively, these data provided convincing evidence that mitochondrial stress also regulates macrophage M2 program implicated in the pathogenesis of pulmonary fibrosis.

Interestingly, there is compelling evidence that endoplasmic reticulum (ER) stress modulates the activation of M2 macrophages [118, 119]. During the past few years, our laboratory has been focused on the effect of ER stress on fibrogenesis. We first demonstrated evidence that pulmonary fibrosis manifests altered C/EBP homologous protein (CHOP) expression and ER stress in both IPF patients and animals with bleomycin-induced pulmonary fibrosis. In consistent with these observations, mice deficient in *Chop* were protected from bleomycin-induced lung injury and fibrosis. Specifically, the loss of *Chop* significantly attenuated TGF-β production along with reduced M2 macrophage infiltration in the lung following bleomycin induction. Mechanistic studies revealed that *Chop* deficiency suppressed the M2 program in macrophages, which then attenuated TGF-β secretion. The loss of *Chop* enhanced the expression of SOCS1 and SOCS3, thereby inhibiting STAT6/PPARγ signaling that is essential for macrophage M2 program [87]. Similarly, an ovalbumin (OVA)-induced allergic airway inflammatory model revealed that Chop regulates STAT6 phosphorylation, thereby enhancing the expression of mouse transcription factor EC (Tfec), which then transcribes IL-4 receptor a (IL-4Ra) expression to promote M2 program in macrophages [120]. Taken together, those data provide novel insights into the role of ER stress in modulating macrophage M2 program implicated in the pathogenesis of pulmonary fibrosis.

## Conclusion

**Fig. 2:**
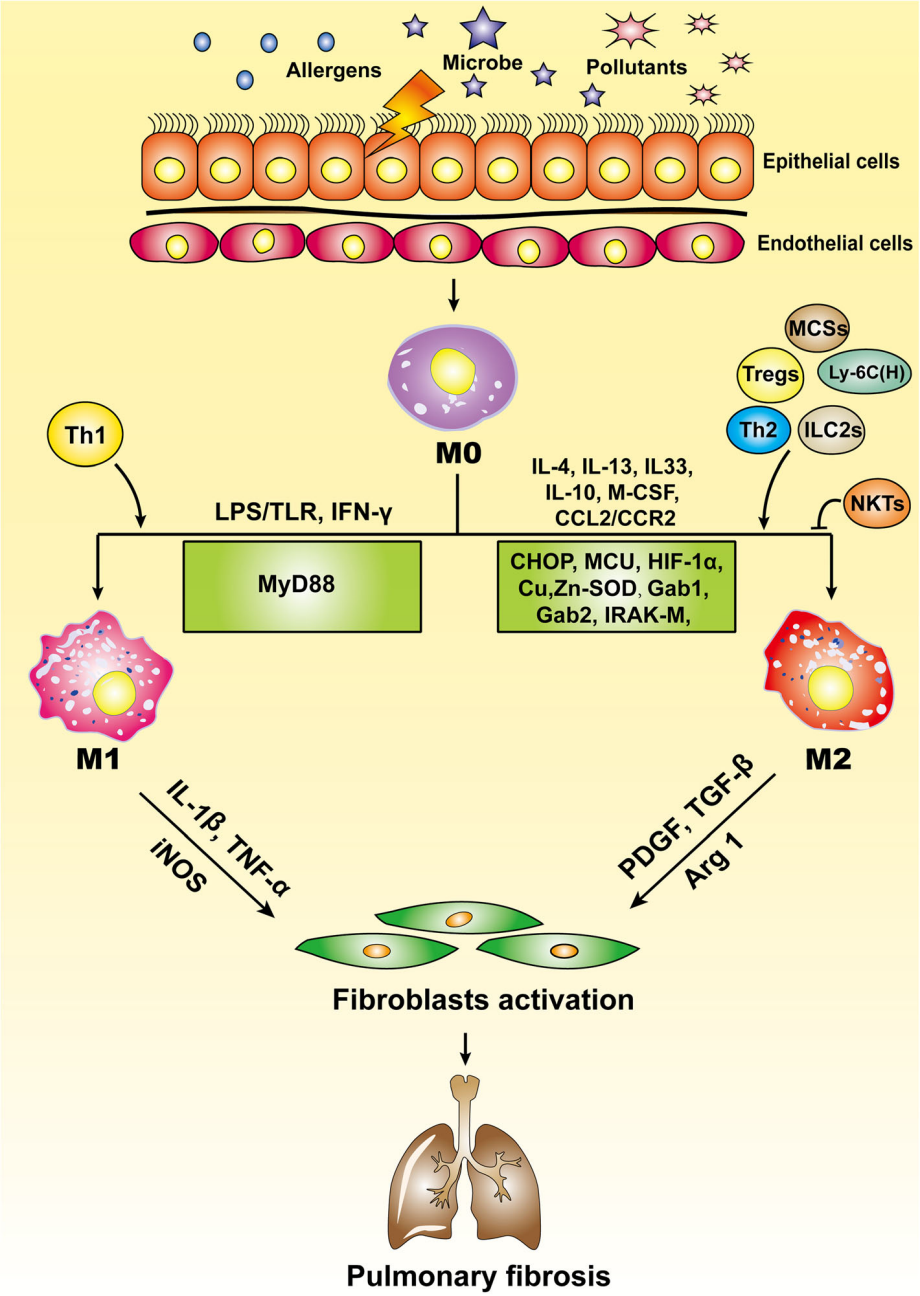
Macrophages in the pathogenesis of pulmonary fibrosis. This schematic diagram demonstrates the mediators for modulation of macrophage subtypes and how macrophages contribute to IPF initiation and progression. M1 macrophages play an essential role for wound healing after alveolar epithelial injury, while M2 macrophages are require to resolve inflammatory responses in the lung, and IPF is a pathological outcome resulted from altered wound healing in response to persistent lung injury. Ly-6C (H), Ly-6C^high^ monocytes; MCS, mesenchymal stem cells; ILC2s, lymphoid type 2 cells; NKTs, natural killer T cells; MCU, mitochondrial calcium uniporter; CHOP, C/EBP homologous protein; IRAK-M, IL-1R–associated kinase-M; Arg 1, arginase 1; iNOS, inducible nitric oxide synthase

Lung M1 and M2 macrophages are distinct cell subtypes and are both involved in the pathogenesis of pulmonary fibrosis. M1 macrophages express high levels of proinflammatory cytokines, while M2 macrophages express high levels of Th2-type cytokines. Owing to their different cytokine expression profiles, M1 and M2 macrophages play different roles in the pathogenesis of pulmonary fibrosis. Generally, M1 macrophages are responsible for wound healing after alveolar epithelial injury, while M2 macrophages are designated to resolve wound healing processes or terminate inflammatory responses in the lung. IPF is a pathological consequence resulted from altered wound healing in response to persistent lung injury. A variety of regulatory cytokines, chemokines, mediators, and immune-regulatory cells affect polarization and chemotaxis of the lung macrophages (Fig. 2). These mediators interplay and influence disease duration and severity through altered polarization of M1 and M2 cells. Therefore, strategies aimed at modulation of lung macrophage phenotypes may have great potential for prevention and treatment of pulmonary fibrosis in clinical settings.

As noted above, IPF is a hard-to-diagnose fatal interstitial lung disease with a poor response to traditional therapies. Therefore, the development of reliable and fast diagnostic techniques and effective therapies is highly desirable, while targeting the activation and recruitment of M2 macrophages could be a viable therapeutic strategy. Indeed, pirfenidone, one of the two currently US Food and Drug Administration (FDA) approved drugs (pirfenidone and nintedanib) for treatment of human IPF, exerts its anti-fibrotic property in part by suppressing TGF-β expression relevant to macrophage M2 polarization and fibroblast activation [121]. Moreover, neotuberostemonine (NTS), one of the traditional Chinese medicines included in all versions of the Chinese Pharmacopoeia, can effectively attenuate BLM-induced lung fibrosis via suppressing the recruitment and polarization of M2 macrophages [122]. Intriguingly, Ji et al. demonstrated that mineralocorticoid receptor (MR) antagonism by liposome-encapsulated spironolactone (Lipo-SP) could alleviate bleomycin-induced acute pulmonary injury and fibrosis, partially by reducing circulating inflammatory Ly6C^high^ monocyte expansion and repressing alternatively activated mononuclear phagocytes in the alveolar compartment [123, 124].

Although therapies for IPF include a variety of drugs and non-pharmacological interventions, there is still a pressing need for new therapeutic approaches as current therapies are unable to effectively attenuate disease progression or reverse lung fibrosis. Despite past extensive studies, many questions remain unsolved regarding the exact mechanisms of manipulating the balance of M1/M2 phenotype in IPF pathogenesis. Future studies aimed at dissecting the interplay between macrophages and fibroblasts and how macrophages create an extracellular milieu in favor of fibroblast activation and myofibroblast proliferation, would be crucial for developing effective therapies against IPF and other fibrotic diseases.

## Abbreviations

ABCA3: ATP binding cassette member A3
AEC II: Type II alveolar epithelial cell
AMs: Alveolar macrophages
ARDS: Acute respiratory distress syndrome
Arg 1: Arginase 1
ATF 7: Activating transcription factor 7
BMDMs: Bone marrow-derived monocytes
Cav-1: Caveolin-1
CHOP: C/EBP homologous protein
ECM: Excessive extracellular matrix
ER: Endoplasmic reticulum
ExM: Exudate macrophages
FDA: Food and drug administration
Fizz1/RELM-α: Found in inflammatory zone 1
GM-CSF: Granulocyte-macrophage colony-stimulating factor
GWASs: Genome-wide association studies
HIF-1α: Hypoxia-inducible factor 1-α
HMGB1: High-mobility group box 1
IL-4Ra: IL-4 receptor a
ILC2s: Innate lymphoid type 2 cells
IMs: Interstitial macrophages
iNOS: Inducible nitric oxide synthase
IPF: Idiopathic pulmonary fibrosis
IRAK-M: IL-1R–associated kinase-M
IRF4: Interferon regulatory factor 4
IRF-5: Interferon regulatory factor 5
Jmjd3: Jumonji domain-containing protein 3
KLF-4: Krueppel-like factor 4
Lipo-SP: liposome-encapsulated spironolactone
LPS: Lipopolysaccharide
M1: Classically activated macrophages
M2: Alternatively activated macrophages
MCU: Mitochondrial calcium uniporter
MIP-1α: Macrophage inflammatory protein-1α
MMP1: Matrix metalloproteinase 1
MMP19: Matrix metalloproteinase 19
MR: mineralocorticoid receptor
MSCs: Mesenchymal stem cells
MUC5B: Mucin 5B gene
NKTs: Natural killer T cells
NO: Nitric oxide
NTS: Neotuberostemonine
PPARγ: Peroxisome proliferator-activated receptor gamma
RTEL1: Regulator of telomere elongation helicase 1
SFTPA2: Surfactant protein A2
SFTPC: Surfactant protein C
SPPL2C: Signal peptide peptidase like 2C
STUB1: STIP1 homology and U-Box containing protein 1
TERC: Telomerase RNA component
TERT: Telomerase reverse transcriptase
Tet: Ten eleven translocation
Tet2: Ten eleven translocation methylcytosine dioxygenase
Tfec: Transcription factor EC
TLR: Toll-like receptor
TOLLIP: Toll interacting protein
Tregs: Regulatory T cells
TSC1: Tuberous sclerosis complex 1
